# Visfatin Serum Levels Predict Mortality in Critically Ill Patients

**DOI:** 10.1155/2018/7315356

**Published:** 2018-08-26

**Authors:** Alexander Koch, Ralf Weiskirchen, Alexander Krusch, Jan Bruensing, Lukas Buendgens, Ulf Herbers, Eray Yagmur, Ger H. Koek, Christian Trautwein, Frank Tacke

**Affiliations:** ^1^Department of Medicine III, RWTH-University Hospital Aachen, Pauwelsstrasse 30, 52074 Aachen, Germany; ^2^Institute of Molecular Pathobiochemistry, Experimental Gene Therapy and Clinical Chemistry, RWTH-University Hospital Aachen, Pauwelsstrasse 30, 52074 Aachen, Germany; ^3^Medical Care Center, Dr. Stein and Colleagues, Mönchengladbach, Germany; ^4^Section of Gastroenterology and Hepatology, Department of Internal Medicine, Maastricht University Medical Centre (MUMC), Maastricht, Netherlands

## Abstract

The adipokine visfatin, also termed pre-B-cell colony-enhancing factor (PBEF), is mainly derived from adipose tissue but has been implicated in the regulation of innate immune responses. We hypothesized that visfatin could be a potential circulating biomarker in critical illness and sepsis. We therefore measured serum levels of visfatin in a cohort of 229 critically ill medical patients upon admission to the intensive care unit (ICU). In comparison to 53 healthy controls, visfatin levels were significantly elevated in medical ICU patients, especially in patients with sepsis. Visfatin serum concentrations were strongly associated with disease severity and organ failure but did not differ between patients with or without obesity or type 2 diabetes. Visfatin levels correlated with biomarkers of renal failure, liver dysfunction, and other adipokines (e.g., resistin, leptin, and adiponectin) in critically ill patients. High visfatin levels at ICU admission indicated an increased mortality, both at the ICU and during long-term follow-up of approximately two years. Our data therefore demonstrate that circulating visfatin is a valuable biomarker for risk and prognosis assessment in critically ill patients. Furthermore, visfatin seems to be involved in the pathogenesis of excessive systemic inflammation, supporting further research on visfatin as a therapeutic target.

## 1. Introduction

Besides their important roles in metabolism, adipocytokines or adipokines, i.e., hormones released from adipose tissue, are increasingly recognized as important regulators of immunity [1]. It has been suggested that adipokines contribute to the excessive systemic inflammatory reaction commonly observed in critical illness. We and others have previously shown that serum levels of the adipokines resistin and adiponectin are significantly elevated in critically ill patients and are associated with patients' mortality [2–6]. Relatively few data exist on visfatin in the setting of critical illness. The adipokine visfatin was initially identified in lymphocytes and is therefore also called pre-B-cell colony-enhancing factor (PBEF) [7]. Leukocytes have been identified as a major source of circulating visfatin [8]. Moreover, visfatin is also involved in activation and attraction of inflammatory cells. Experimental data obtained from human cells and mouse models revealed that visfatin is a chemoattractant for neutrophils [9], promotes neutrophil survival [10], and induces the dose-dependent release of cytokines in monocytes [11]. Interesting findings obtained from smaller trials demonstrated elevated visfatin serum levels in patients with respiratory diseases [12–14] and neonatal sepsis [15] as well as in patients with severe trauma or with critical neurological diseases [2]. Based on these findings, we analyzed circulating visfatin levels in a large cohort of 229 prospectively enrolled critically ill patients at our medical intensive care unit (ICU) in order to define the potential pathogenic role of visfatin in critical illness and its utility as a clinical biomarker in the ICU setting.

## 2. Materials and Methods

### 2.1. Study Design and Patient Characteristics

Critically ill patients were included at admission to the medical ICU at the University Hospital Aachen, Germany. Patients, who were admitted for postinterventional observational stay or underwent an elective procedure, were excluded [16]. The local ethics committee approved our study in accordance to the ethical standards laid down in the Declaration of Helsinki (reference number EK 150/06). The patients were categorized as sepsis and nonsepsis according to the “Third International Consensus Definitions for Sepsis and Septic Shock (Sepsis-3)” [17] and were treated following the current guidelines for treatment of sepsis (Surviving Sepsis Campaign) [18]. As a healthy control group, we analyzed blood donors (36 male, 17 female, median age 37 years, range 25–67 years, BMI median 25 kg/m^2^, range 19–34 kg/m^2^) with normal blood counts, normal values of liver enzymes, and a negative serology for viral hepatitis and HIV [19].

In order to determine long-term outcome, we contacted the patients, their relatives, and/or the general practitioner in approximately 6-month intervals after discharge from hospital for two years [19].

### 2.2. Measurements of Visfatin and Adipokines

Blood samples were collected at the time of admission (before specific therapeutic measures had been started at the ICU) and centrifuged, and serum was stored at −80°C. Visfatin was analyzed with a commercial ELISA kit (USCN Life Science, #E90638Hu, BIOZOL Diagnostica, Eching, Germany). Measurements of the other adipocytokines and related proteins resistin, adiponectin, leptin, and leptin receptor were included as previously reported [3, 4, 20].

### 2.3. Statistical Analysis

Due to the high range of visfatin values, especially comparing healthy controls and critically ill patients, all visfatin serum concentrations are presented as logarithmic values. The Mann-Whitney *U*-test was used to test differences between the two groups; correlations were tested according to Spearman's rank correlation method. All values, including outside values as well as far out values, were included. *p* values less than 0.05 were considered as statistically significant.

The prognostic value of visfatin on the outcome was evaluated by Cox regression models. Survival curves were generated by Kaplan-Meier analyses with a visfatin cutoff level calculated via the Youden Index [21]. All analyses were performed with IBM SPSS Statistics (SPSS; Chicago, Illinois).

## 3. Results

### 3.1. Visfatin Serum Levels Are Significantly Elevated in Critically Ill Patients as Compared with Healthy Controls

**Figure 1:**
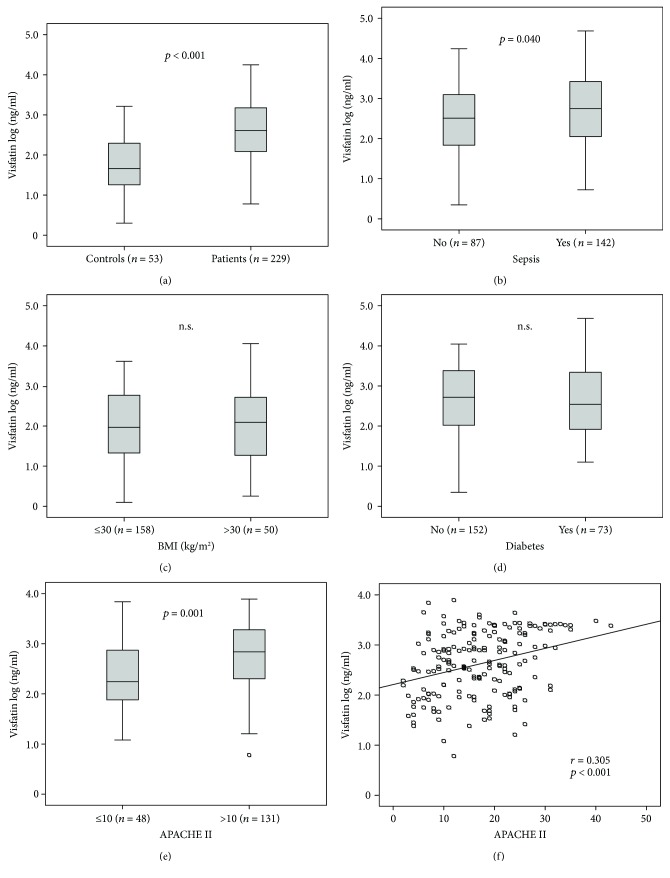
Visfatin levels in critically ill patients. (a) Visfatin serum concentrations (displayed as log visfatin) are significantly elevated in critically ill patients compared with controls. (b–e) Subgroup analyses of visfatin levels in critically ill patients, according to sepsis (b), obesity (c) (defined by body mass index (BMI) above 30 kg/m^2^), diabetes (d), or disease severity (APACHE II score above 10). (f) Visfatin levels correlate with APACHE II score in critically ill patients.

Visfatin serum levels were measured in a prospectively recruited cohort of 229 critically ill medical patients. Visfatin serum concentrations were approximately one log-fold higher in critically ill patients (median visfatin log 2.61 ng/ml, range 0.78–4.25, Table 1) compared to healthy controls (*n* = 53, median visfatin log 1.66 ng/ml, range 0.30–3.21, *p* < 0.001; Figure 1(a)). Visfatin levels did not correlate with the age, neither in patients (*r* = 0.24, *p* = 0.723) nor in healthy controls (*r* = 0.101, *p* = 0.474). Of the 229 ICU patients, 142 were admitted due to sepsis, while 87 patients had a critical illness due to other origin such as cardiopulmonary, gastrointestinal, or hepatic disorders (Table 2). Patients with sepsis had further elevated visfatin levels compared to nonsepsis ICU patients (visfatin log 2.70 ng/ml versus 2.51 ng/ml, *p* = 0.04; Figure 1(b)). Within the sepsis patients, the site of infection (e.g., pneumonia, bloodstream, abdominal, and urogenital) did not affect visfatin concentrations.

### 3.2. Diabetes or Obesity Did Not Impact Visfatin Levels at Admission to the ICU

As high visfatin levels have been consistently associated with obesity, type 2 diabetes, and the metabolic syndrome [7, 22, 23], we tested whether obesity or type 2 diabetes as a comorbidity at ICU admission impacted visfatin levels. Unexpectedly, neither obesity as defined by a body mass index (BMI) above 30 kg/m^2^ (Figure 1(c)) nor preexisting type 2 diabetes (Figure 1(d)) was associated with visfatin serum concentrations. Moreover, serum glucose at ICU admission or glycosylated haemoglobin A1 (HbA1c) did not correlate with visfatin levels in critically ill patients (data not shown). In addition, *n* = 23 patients admitted to the ICU had preexisting liver cirrhosis. Their visfatin levels (median log visfatin 2.88, range 1.82–3.74) did not differ significantly from ICU patients without liver cirrhosis (median log visfatin 2.57, range 0.78–4.25, *p* = 0.151).

### 3.3. Visfatin Serum Concentrations Are Strongly Associated with Disease Severity

Based on our finding of high levels of visfatin in ICU patients, we next tested the potential association of visfatin with the severity of critical illness. In fact, patients with an acute physiology and chronic health II [APACHE II] score above 10 displayed significantly higher visfatin serum levels than patients with APACHE II values below or equal to 10 (Figure 1(e)). Moreover, visfatin levels directly correlated with APACHE II scores (*r* = 0.305, *p* < 0.001; Figure 1(f)), sequential organ failure assessment (SOFA), or simplified acute physiology score 2 (SAPS2) scores (Table 3).

### 3.4. Visfatin Levels Are Correlated with Biomarkers of Renal Failure, Liver Failure, and Metabolic Disturbances in Critically Ill Patients

**Figure 2:**
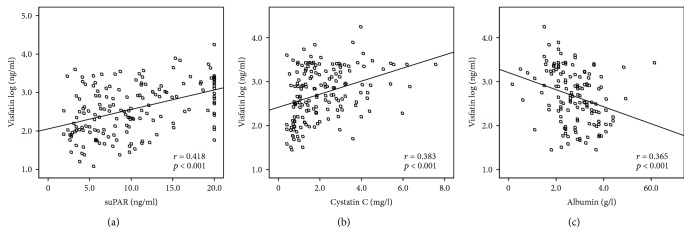
Visfatin levels correlate with inflammation and organ failure. (a–c) Correlation analyses revealed associations between serum visfatin and biomarkers of systemic inflammation (e.g., soluble urokinase plasminogen activator receptor (suPAR)) (a), renal failure (e.g., cystatin) (c, b), or hepatic dysfunction (e.g., albumin) (c).

Due to the well-established role of circulating visfatin in systemic inflammation and cytokine release [24], we analyzed correlations of visfatin in ICU patients with various biomarkers of inflammation, organ dysfunction, and metabolism (Table 3). Visfatin concentrations correlated closely with markers of inflammation including C-reactive protein, procalcitonin, interleukin-6 (IL-6), and other cytokines (Table 3), confirming observations obtained in neonatal sepsis [15]. Visfatin also correlated with soluble urokinase plasminogen activator receptor (suPAR, Figure 2(a)), a prognostic biomarker of inflammation in the ICU setting [25]. Circulating visfatin displayed a close association with renal dysfunction, as indicated by several markers including creatinine, cystatin C (Figure 2(b)), and their glomerular filtration rates (Table 3). Similar results were noted for markers reflecting liver function like albumin (Figure 2(c)), bilirubin, and coagulation factors (Table 3). Visfatin levels correlated with the other adipocytokines and related proteins assessed in our cohort, namely, leptin, leptin receptor, adiponectin, and resistin (Table 3).

### 3.5. High Visfatin Serum Concentrations at ICU Admission Are Associated with Adverse Prognosis

**Figure 3:**
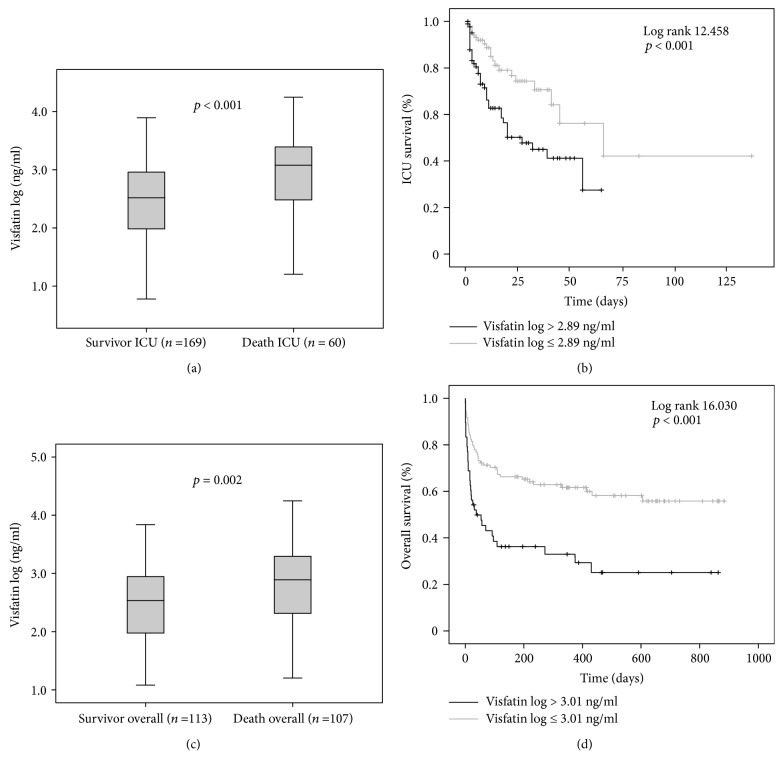
Visfatin is a biomarker for mortality in critically ill patients. (a) At the time of ICU admission, patients that died during the course of ICU treatment had significantly higher serum visfatin levels than survivors (*p* < 0.001). (b) Patients with high or low visfatin levels displayed different ICU mortalities by Kaplan-Meier survival curve analysis. (c) A similar observation was obtained when visfatin levels at ICU admission were compared between patients that died during the total observation period and survivors (*p* = 0.002). (d) High visfatin levels at ICU admission predicted the overall mortality during long-term follow-up in critically ill patients (Kaplan-Meier survival curve analysis for the optimal visfatin cutoff is displayed).

In critically patients, who subsequently died during the ICU treatment (*n* = 60), we found significantly elevated visfatin levels at admission to the ICU (Figure 3(a)), suggesting that visfatin might serve as a prognostic biomarker in critical diseases. In fact, Cox regression analysis revealed that visfatin was a robust predictor of ICU mortality (*p* < 0.001). Kaplan-Meier curves were calculated with a cutoff value of log visfatin 2.89 ng/ml that showed the optimal ratio of sensitivity and specificity for mortality using the Youden Index. Here, visfatin levels clearly discriminated between survivors and nonsurvivors (Figure 3(b)).

Even patients that are successfully discharged from the ICU have a tremendous risk of mortality during the first years of follow-up [26]. We were able to assess long-term survival in 220 out of the 229 patients. Visfatin levels at ICU admission were significantly higher in patients that died during the follow-up period of approximately two years compared with survivors (Figure 3(c)). Cox regression analysis confirmed the prognostic value of visfatin as a predictor of long-term mortality (*p* = 0.001). Using the calculated optimal cutoff (log visfatin 3.01), patients with high visfatin demonstrated an unfavourable outcome, as depicted by Kaplan-Meier survival curve analysis (Figure 3(d)). The validity and performance of visfatin as a biomarker for the prediction of ICU or overall survival in critically ill patients are summarized in Table 4.

Notably, visfatin levels appeared more suited to predict outcome in comparison to other adipocytokines. By receiver operating characteristics (ROC) curve analyses, visfatin levels reached an area under the curve (AUC) to predict ICU mortality of 0.687, while resistin (0.562), adiponectin (0.623), leptin (0.404), and leptin receptor (0.580) demonstrated lower values. For overall mortality, visfatin reached a higher AUC of 0.686 compared to resistin (0.563), adiponectin (0.638), leptin (0.407), and leptin receptor (0.609).

## 4. Discussion

The dysregulation of adipocytokines has been widely noted in critical illness and linked to systemic inflammation. Among interesting candidates of adipokines as biomarkers, leptin, adiponectin, and resistin have been thoroughly investigated [1–4, 20]. In this study, we focused on visfatin, an adipocytokine with several metabolic but also inflammation-orchestrating functions [24]. In a large cohort of prospectively enrolled critically ill medical patients, we demonstrate that visfatin serum levels are highly elevated compared to controls, associated with sepsis and disease severity, correlated to organ dysfunction, and, most importantly, serve as a reliable predictor of mortality. Our findings are well in agreement with smaller trials reporting elevated visfatin and the association with poor outcome in patients with respiratory diseases [12–14] and neonatal sepsis [15]. Similar findings have also been reported from patients with severe trauma or with critical neurological diseases [2].

The close association between high visfatin levels and increased short- or long-term mortality in our study may be well explained by the strong correlations between visfatin and inflammatory mediators and cytokines, disease severity (e.g., clinical scores), and biomarkers reflecting organ failure. However, there is increasing evidence emerging that visfatin is directly involved in the pathogenesis of critical illness and systemic inflammation. Visfatin was found to be a chemoattractant for neutrophils [9] and has direct effects on neutrophil survival [10], which could jointly promote excessive release of cytokines [24], production of oxidative stress factors, and subsequently result in tissue damage and organ failure [2]. In support of this hypothesis, the experimental inhibition of visfatin in mouse models of ventilator-associated lung injury reduced neutrophil infiltration, organ injury, and mortality [9]. Moreover, distinct single-nucleotide polymorphisms (SNPs) in the visfatin gene have been identified in humans [27, 28], of which the SNP −1543T was linked to a reduced risk of mortality, while the SNP −1001G was associated with a higher risk of mortality in patients with acute respiratory distress syndrome [29].

In our cohort, 24% of the critically ill medical patients were obese or morbidly obese, as defined by a BMI above 30 kg/m^2^. This is in line with observations in the United States, where at least 25% of adult ICU patients are overweight, obese, or morbidly obese [30, 31]. Interestingly, we did not find dysregulated visfatin levels between ICU patients with or without obesity, supporting that circulating visfatin levels in critical illness are primarily attributable to the extent of inflammation and not adiposity itself. Nonetheless, visfatin levels were closely correlated with adiponectin, resistin, and (inversely) leptin, indicating a concerted yet rectified activation of adipose tissue inflammation [1].

As outcome prediction is of major interest in the ICU setting, there is a high medical need to complement current prognostic models (e.g., APACHE II, SAPS, and SOFA) by additional biomarkers that could indicate the long-term prognosis beyond the acute critical illness [32]. Visfatin demonstrated in our study an exceptional value to predict the overall mortality during a two-year follow-up period. Thus, our data indicated that visfatin could be possibly used, either alone or in combination with other adipokines, for a more accurate prognostication in critical illness.

## 5. Conclusions

We demonstrate in our study comprising 229 critically ill medical patients that circulating levels of the adipokine visfatin were significantly elevated at admission to the ICU, as compared with healthy controls. Visfatin serum concentrations were strongly associated with disease severity, organ failure, and sepsis, but not with obesity or type 2 diabetes. High visfatin levels at ICU admission indicated an increased mortality, both at the ICU and during long-term follow-up. Further research should aim at implementing visfatin as a prognostic biomarker in a comprehensive risk assessment algorithm at the ICU. Moreover, the close association between visfatin and prognosis as well as experimental data on visfatin neutralization in animal models supports to explore visfatin as a therapeutic target in excessive systemic inflammation and sepsis.

## Figures and Tables

**Table 1:** Baseline patient characteristics and visfatin serum measurements.

Parameter	All patients	Nonsepsis	Sepsis
Number	229	87	142
Sex (male/female)	133/96	51/36	82/60
Age median (range) (years)	63 (18–90)	61 (18–85)	64 (20–90)
APACHE II score median (range)	16 (2–43)	14.5 (2–33)	18 (3–43)
ICU days median (range)	7 (1–137)	5 (1–45)	9.5 (1–137)
Death during ICU *n* (%)	60 (26%)	15 (17%)	45 (32%)
Death during follow-up (total) *n* (%)	107 (47%)	31 (36%)	76 (54%)
Mechanical ventilation *n* (%)	157 (69%)	53 (61%)	104 (73%)
Preexisting diabetes *n* (%)	73 (32%)	27 (31%)	46 (32%)
Preexisting cirrhosis *n* (%)	23 (10%)	16 (18%)	7 (5%)
BMI median (range) (m^2^/kg)	25.9 (15.9–86.5)	25.5 (15.9–53.9)	26.0 (17.1–86.5)
WBC median (range) (×10^3^/*μ*l)	12.8 (0–149)	12.0 (1.8–29.6)	14.0 (0–149)
CRP median (range) (mg/dl)	92 (5–230)	17 (5–230)	153 (5–230)
Procalcitonin median (range) (*μ*g/l)	0.7 (0.03–207.5)	0.2 (0.03–100)	2.3 (0.10–207.5)
Creatinine median (range) (mg/dl)	1.35 (0.1–21.6)	1.0 (0.2–15.0)	1.7 (0.1–21.6)
INR median (range)	1.18 (0.9–13)	1.17 (0.9–6.7)	1.18 (0.9–13)
Log visfatin median (range) (ng/ml)	2.61 (0.78–4.25)	2.51 (0.78–3.89)	2.70 (1.08–4.25)

For quantitative variables, median and range (in parentheses) are given. APACHE: acute physiology and chronic health evaluation; BMI: body mass index; CRP: C-reactive protein; ICU: intensive care unit; INR: international normalized ration; WBC: white blood cell.

**Table 2:** Disease etiology of the study population leading to ICU admission.

	Sepsis	Nonsepsis
	142	87
Etiology of sepsis critical illness		
Site of infection *n* (%)		
Pulmonary	82 (58%)	
Abdominal	26 (18%)	
Urogenital	4 (3%)	
Other	30 (21%)	
Etiology of nonsepsis critical illness *n* (%)		
Cardiopulmonary disorder		29 (33%)
Acute pancreatitis		11 (13%)
Acute liver failure		4 (5%)
Decompensated liver cirrhosis		15 (17%)
Severe gastrointestinal hemorrhage		6 (7%)
Nonsepsis other		22 (25%)

**Table 3:** Correlations with visfatin (log) serum concentrations at ICU admission (Spearman rank correlation test, only significant results are shown).

Parameters	ICU patients	
	*r*	*p*
*Disease severity*		
APACHE II score	0.305	<0.001
SOFA score	0.494	<0.001
SAPS2 score	0.406	<0.001
*Inflammation*		
C-reactive protein	0.256	<0.001
Procalcitonin	0.379	<0.001
suPAR	0.418	<0.001
White blood cell count	0.131	0.048
Interleukin-6	0.291	<0.001
TNF	0.331	0.003
Interleukin-10	0.423	<0.001
*Renal function*		
Creatinine	0.421	<0.001
GFR (creatinine)	−0.427	<0.001
Cystatin C	0.383	<0.001
GFR (cystatin C)	−0.372	<0.001
Urea	0.377	<0.001
Uric acid	0.231	<0.001
*Liver function*		
Protein	−0.352	<0.001
Albumin	−0.365	<0.001
Pseudocholinesterase	−0.316	<0.001
Bilirubin	0.167	0.012
Bilirubin (conjugated)	0.212	0.009
Alkaline phosphatase	0.218	0.001
AST	0.196	0.004
INR	0.315	<0.001
Prothrombin time	−0.336	<0.001
aPTT	0.283	<0.001
D-dimers	0.380	<0.001
Antithrombin III	−0.456	<0.001
Fibrinogen	−0.385	<0.001
*Metabolism*		
Leptin	−0.340	0.001
Leptin receptor	0.318	0.002
Adiponectin	0.235	0.02
Resistin	0.313	0.002

APACHE: acute physiology and chronic health evaluation; aPTT: activated prothrombin time; AST, aspartate aminotransferase; GFR: glomerular filtration rate; INR: international normalized ratio; SAPS: simplified acute physiology score; SOFA: sequential organ failure assessment; suPAR: soluble urokinase plasminogen activator receptor; TNF: tumor necrosis factor.

**Table 4:** Serum visfatin (log) performance as a biomarker to predict ICU or overall mortality.

	ICU mortality	Overall mortality
Visfatin (log) optimal cutoff	2.8882	3.0094
Sensitivity	0.63	0.45
Specificity	0.69	0.80
Positive predictive value	0.42	0.68
Negative predictive value	0.84	0.60
Youden Index	0.32	0.25
LHR+	2.02	2.20
LHR−	0.53	0.69
Diagnostic odds ratio	3.77	3.18

LHR: likelihood ratio.

## Data Availability

The data used to support the findings of this study are available from the corresponding author upon request.
